# P286: Decreasing nosocomial exposures of tuberculosis to healthcare workers at emergency department in a tertiary care hospital in Karachi, Pakistan

**DOI:** 10.1186/2047-2994-2-S1-P286

**Published:** 2013-06-20

**Authors:** SA Musani, RR Essani, RM Khowaja, NR Alwani, S Lakhani, A Zafar

**Affiliations:** 1 Infection Control Committee, The Aga Khan University Hospital, Karachi, Pakistan; 2 Emergency Department, The Aga Khan University Hospital, Karachi, Pakistan

## Introduction

Exposure to Tubercolusis is a known risk to health care workers (HCWs)[1]. Recent studies have reported estimated incidence rate of 7.2% for Latent TB and 1,180/100,000 for TB infection among HCW.[2] Risk of exposure to TB is higher in certain work locations including Endoscopy units. TB laboratory, TB clinics, and emergency departments (ED) due to lack of traige and delay in diagnoses.

## Objectives

The aim of this study was to limit exposure of HCWs in ED.

## Methods

Retrospective study of exposed HCWs from January till July 2012 through descriptive analyses was done. Post intervention effectiveness was measured from September 2012 –February 2013.

## Results

A total of 132 HCWs were exposed to 19 TB patients in seven months (mean = 19 HCWs/month and range 0 –48 HCWs/ month), out of which 53 (40%) of the exposed staff belonged to ED, thus Infection control nurses (ICNs) initiated focused rounds in ED. The rounds focused on all suspected TB patients; and on the spot facilitation for triage to prevent delay in diagnoses, timely initiation of isolation precautions and speeding up for admission process was done.

After the implementation of focused round by ICNs it was analyzed that the overall exposure of HCWs to tuberculosis decreased from 132 to two (02) HCWs who were exposed to 13 patients in the period of September 2012 –February 2013. Furthermore, after the intervention the exposure rate of ED also decreased from 40% to zero (0%). The added benefit of this intervention also supported the institution in terms of decreasing the financial burden caused by expenses incurred for post-exposure screening and prophylaxis.

## Conclusion

A significant reduction in the exposures was observed through initiation of a simple intervention of assessing, facilitation and timely communication of suspected TB cases admitted through ER.

## Competing interests

None declared
